# Comparative analysis of clinical image evaluation charts for panoramic radiography

**DOI:** 10.1007/s11282-024-00765-3

**Published:** 2024-07-08

**Authors:** Yeonhee Kim, Samsun Lee, Gyudong Jo, Ahyoung Kwon, Juhee Kang, Joeun Kim, Kyunghoe Huh, Wonjin Yi, Minsuk Heo, Soonchul Choi

**Affiliations:** Department of Oral and Maxillofacial Radiology and Dental Research Institute, School of Dentistry, Seoul National University, Seoul, South Korea

**Keywords:** Panoramic radiography, Simple clinical image evaluation chart, Professional clinical image evaluation chart, Image evaluation

## Abstract

**Objective:**

To compare and analyze professional (P chart) and simple (S chart) clinical image evaluation charts for evaluating panoramic radiograph image quality.

**Methods:**

Ten evaluators assessed 285 clinical panoramic radiograph images. The evaluators were divided into oral and maxillofacial radiologists (OMFR, n = 5) and general dentist (dentists not specializing in oral and maxillofacial radiology, G, n = 5) groups. For image evaluation, P and S charts provided by the Korean Academy of Oral and Maxillofacial Radiology were used. Scores of items for each evaluation chart were used to compare the reliability, correlation, evaluation scores, evaluation time, and preference, and statistical analyses were performed using IBM SPSS Statistics.

**Results:**

The S chart showed similar levels of evaluation scores at shorter evaluation time, as compared to the P chart. In the results for each evaluation chart, all analyzed correlations were statistically significant. Total score, image density/contrast/sharpness, and overall image quality items showed a very high positive correlation in the P chart. While the overall range of correlation coefficients was relatively lower in the S chart than the P chart, the same items showed high correlation coefficients. In the preference evaluation, both the professional and generalist groups preferred the S chart.

**Conclusions:**

A comparative analysis with the P chart, revisions, and upgrades are needed for the S chart items that showed low correlations in this study, such as artifacts, coverage area, and patient movement.

## Introduction

Panoramic radiography is a radiological imaging technique that is used to view the overall morphology of facial structures, including the teeth, periodontal tissues, and upper/lower jawbones, in a single image. This technique is commonly used for screening patients who visit a dental hospital for the first time and for diagnostic purposes in patients who present with symptoms [1, 2]. Panoramic radiography equipment consists of a system that combines the principles of tomography and scanning. Consequently, images with poor diagnostic value may be obtained when the target jawbone is not accurately positioned on the focal trough (upper layer) or due to patient positioning and mechanical errors [3].

With respect to quality control for panoramic radiography in Korea, the “Rules for Safety Management of Diagnostic Radiation Emitting Generators” stipulate that dental diagnostic X-ray equipment must undergo regular inspections every three years after the initial installation [4]. The X-ray tubes, control devices, and high-voltage generators are assessed in these inspections; however, standards for clinical image quality (of panoramic radiography) have not been established. At present, quality control testing of medical imaging data obtained by computed tomography systems, magnetic resonance imaging systems, and mammography devices is conducted systematically by the Korean Institute for Accreditation of Medical Imaging (KAMI) to improve the quality of medical images [5]. However, panoramic radiography systems are not included in the testing by KAMI, and as a result, a quality control system for image quality has not been established.

A previous study used evaluation charts to evaluate the image quality of panoramic radiography [6–9]. Moreover, Choi et al. assessed digital panoramic radiographs using image quality evaluation charts and a phantom stand [10]. In a separate study, researchers investigated the clinical imaging quality of panoramic radiography images in Korean dental clinics [11]. In this study, the overall level of panoramic radiographs in Korea was found to be above average, and patient positioning and density/resolution/contrast were identified as the key factors that affected the image quality.

Outside Korea, attempts have been made to develop and implement guidelines to evaluate the image quality in panoramic radiography. For example, in a study published in the British Dental Journal in 1999, Ruston et al. evaluated the image quality of a total of 1813 panoramic images from 41 general dental clinics [12]. Although no specific image quality evaluation table was used, 0.8% of the videos were rated as “excellent,” 66.2% were rated as “diagnostically acceptable,” and 33% were rated as "unacceptable." At present, the UK provides the most specific standards for the quality of panoramic radiological images. For example, European guidelines recommend the following to be evaluated: (1) patient preparation/instruction is adequate, (2) no patient positioning errors, (3) correct anatomical coverage, (4) good density and contrast, (5) no cassette/screen problems, and (6) adequate processing and darkroom techniques [13]. The guidelines also mention that the image quality should be evaluated as “Excellent” (no faults), “Acceptable” (some faults but not affecting image interpretation), and “Unacceptable” (faults leading to the radiograph being unsuitable for interpretation) and that it is necessary to record the reasons for grading a radiograph of unacceptable quality. In the United States, the Conference of Radiation Control Program Directors (CRCPD) specifies the test items, cycles, and steps through an image quality control manual [14]. However, the section on image quality contains just a single item, which requires the subjective response of the evaluator to the question “Is the image quality maintained at the desired level?” with no other objective indicators. In 2005, the National Council on Radiation Protection and Measurements (NCRP) published a report that mentions the need for quality control for digital radiation equipment in addition to topics related to equipment care, film development, image receptors, darkroom management, a lead apron, thyroid collar, documentation, and image quality control processes [15].

To ensure that images with a high level of diagnostic value can be obtained through panoramic radiography, it is necessary to implement a continuous organic cycle of image quality evaluation and improvement accordingly. As a part of this effort, the Korean Academy of Oral and Maxillofacial Radiology (KAOMFR) developed and has been using the professional evaluation chart (P chart) [6]. Although the P chart has demonstrated high accuracy and favorable results in many studies [6, 16–23], it is difficult to implement for non-radiologists. As an alternative, KAOMFR has developed a simple clinical image quality evaluation chart (S chart) for panoramic radiology that can be used easily even by non-oral and maxillofacial radiologists at regular dental hospitals and clinics [7]. Despite the creation of the S chart, no studies have evaluated its use. Therefore, we aimed to (1) compare the P and S charts based on the correlation analysis, (2) compare the results between OMFR and G, and (3) analyze the preferences for each evaluation chart.

Image quality management for panoramic radiography should be considered an important topic worldwide. It is not only essential for increasing the benefits of radiographic examination but also contributes to ensuring that all patients receive high-quality medical services. Therefore, in this paper, we introduce and compare the P and S charts used in Korea to provide useful information for dental radiography experts and dentists worldwide.

## Materials and methods

### Materials

#### Image data and participants

The Institutional Review Board of [BLINDED FOR REVIEW] approved the analysis of images from a previous study [7]. Personally identifiable information has been omitted. A total of 285 panoramic images were analyzed in this study.

The panoramic radiographs were evaluated by two groups (the OMFR group [oral and maxillofacial radiologists] and G group [dentists not specializing in oral and maxillofacial radiology]) with five evaluators per group. For the OMFR group, 20 oral and maxillofacial radiologists with 4 to 30 years of experience were asked to participate in the study and five specialists who voluntarily consented to participate in the study were enrolled. For the G group, we posted an announcement explaining the objective and analysis methods of the study on the internet and five general dentists with 1 to 3 years of experience voluntarily consented to participate in the study.

#### Evaluation charts

For the evaluation, P and S charts provided by KAOMFR were used. Since the S chart did not have scores assigned to each item, points were assigned to each item (as close as possible to the P chart) for fair comparison with the P chart. The scores assigned to each item of the P chart were as follows: identification (12 points), artifacts (4 points), coverage area (6 points), patient positioning and movement (22 points), image density/contrast/sharpness (46 points), and overall image quality grade (10 points). For comparison with the S chart, patient positioning and movement was separated into patient positioning (18 points) and patient movement (4 points) (Table 1). The scores assigned to each item of the S chart were as follows: identification (12 points), coverage area (6 points), artifacts (4 points), patient movement (4 points), head positioning error (18 points), image density and contrast (46 points), and the overall image quality grade (10 points) (Table 2). All evaluators assigned 12 points to the first evaluation item in the charts (identification, which was not collected to protect personal information).

**Table 1:** Professional clinical image quality evaluation chart of dental panoramic radiography

Evaluation item	Details	Yes	No
1. Identification	Mark for left or right	2	0
	Study Date & Time	2	0
	Name	2	0
	Sex	2	0
	Age	2	0
	Registration number	2	0
2. Artifacts	Internal artifacts or artifacts of unknown origin (record any stains, scratches, static electricity, or detector error, etc.): Not present/ present	2	0
	Artifacts caused by external factors (the patient’s earrings, removable prosthesis, etc.): Not present/ present	2	0
3. Coverage area	1. Temporomandibular joint	6	3/0
	2. Mandibular angle and inferior border of mandible		
	3. Inferior border of the orbit: satisfies 1, 2, 3/satisfies two of all /satisfies 1 or nothing		
4–1. Patient positioning	Correct positioning of jaws on the image focal trough (layer): Adequate/out of image focal trough but diagnosable/unsuitable for diagnosis	4	2/0
	Occlusal plane: Adequate/flat/inverted V or V shape	6	3/0
	Right-left symmetry: Symmetric/the discrepancy is less than 1/2 of the width in M-D of mandibular 1st molar/over 1/2 of the width in M-D of the mandibular 1st molar	4	2/0
	Blurring of anterior region due to overlapping of spinal column: not present/present but does not interfere with diagnosis/unsuitable for diagnosis	4	2/0
4–2. Patient movement	Patient movement—Continuity of anatomic structures: Continuity/step sign under 2 mm/step sign over 2 mm	4	2/0
5. Density, contrast and resolution or sharpness of image	Ability to distinguish between the enamel and dentin: Almost distinguishable/indistinguishable in 2/6 of the region/indistinguishable in 4/6 of the region	6	3/0
	Ability to observe alveolar bone in the alveolar crest: Almost clear/partially clear in 2/6 of the regions/not clear in 4/6 of the region	4	2/0
	Distinguishable PDL space and lamina dura: Almost distinguishable/indistinguishable in 2/6 of the region/indistinguishable in 4/6 of the region	4	2/0
	Accuracy of root shape: Almost clear/partially clear in 2/6 of the region/not clear in 4/6 of the region	4	2/0
	Metal artifact: Distinguishable with secondary caries/indistinguishable in 2/6 of the region/indistinguishable in 4/6 of the region	4	2/0
	Distinguishable the trabecular pattern in alveolar bone: Almost distinguishable/indistinguishable in 2/6 of the region/indistinguishable in 4/6 of the region	4	2/0
	Overall image contrast: Adequate/partially inadequate/almost inadequate	6	3/0
	Overall image density: Homogeneous/partially inhomogeneous/almost heterogeneous	6	3/0
	Overall image sharpness or resolution: Clear/partially blurred/almost not clear	6	3/0
	Noise: Not present/present	2	0
6. Overall image quality grade by expert	Optimal for obtaining diagnosis information/adequate for diagnosis/poor, but diagnosable/unrecognizable, too poor for diagnosis	10	8/6/0
Total score		100	

*PDL* periodontal ligament

**Table 2:** Simple clinical image quality evaluation chart of dental panoramic radiography

Evaluation item	Details	Yes	No
001. Identification	Image acquisition date	2	0
	Registration number	2	0
	Name	2	0
	Age and gender	2	0
		2	0
	Marking for left/right orientation	2	0
	Imaging institution	2	0
2. Coverage area (6)	All areas being observed are included (left and right jawbones; left and right mandibular angle and inferior border of mandible; left and right inferior border of the orbit)	6	3/0
3–1. Artifacts (4)	There must be no artifacts caused by internal or external factors	4	2/0
3–2. Patient movement (4)	Artifacts or phase distortion due to patient movement must not be severe	4	2/0
4. Head positioning error (18)	Enlargement or reduction of maxillary and mandibular anterior regions must not be severe	4	2/0
	Occlusal plane must be adequate	6	3/0
	Left–right symmetry must be adequate	4	2/0
	Blurring due to overlapping must not be severe	4	2/0
5. Density and contrast (46)	Density and contrast must be adequate	12	6/0
	Inferior border of the maxillary sinus must be clearly visible	8.5	4.25/0
	Mandibular canal must be clearly visible	8.5	4.25/0
	Lamina dura and PDL space must be clearly visible	8.5	4.25/0
	Trabecular bone must be clearly visible	8.5	4.25/0
6. Overall image quality grade (10)	Optimal for obtaining diagnosis information/adequate for diagnosis/poor, but diagnosable/unrecognizable, too poor for diagnosis	10	8/6/0

Each item was classified as good, average, or poor, and the corresponding score was assigned

#### Preference survey

All evaluators participated in a survey to assess their chart preference and the reasons for their preference (Table 3).

**Table 3:** Questionnaire to assess the evaluators’ clinical image quality evaluation chart preference

Greetings We are conducting this questionnaire survey to investigate your preference as a part of our comparative analysis on clinical image quality evaluation charts for panoramic radiography. Please take some time from your busy schedule to complete this survey
1. Which of the following applies to you? ① Majored in oral and maxillofacial radiology ② Did not major in oral and maxillofacial radiology
2. Which of the two, P or S chart, do you prefer to use in clinical practice? ① P chart ② S chart
3. Please check all reasons for preferring the evaluation chart that you selected (you may choose more than one) ① Evaluation results are accurate ② Selection of evaluation items is clear ③ Evaluation items are detailed ④ It is easy to select the evaluation items ⑤ Evaluation items are simple ⑥ Can save time, resulting in a shorter evaluation time ⑦ Others
- Thank you for your participation. -

### Data analysis

Each panoramic radiograph collected for the data analysis was assigned a unique identifier number (1–285) to make an order of panoramic radiographs. The OMFR and G groups, each consisting of five members, were divided further into A and B groups: OMFR-A (n = 3), OMFR-B (n = 2), G-A (n = 2), and G-B (n = 3) groups. Each evaluator recorded the evaluation time and score for 285 randomly mixed panoramic radiographs. In this step, the A groups recorded the scores using the P chart and then S chart, while the B groups recorded the scores using the S chart and then P chart to minimize the errors occurring due to differences in the evaluation standards of the evaluators. Each evaluator repeated another round of evaluation of all the items 7 days later and the results from the two rounds of evaluation were used to check the reliability of the evaluation scores. The number of images evaluated by each evaluator per day was limited to ≤ 50 to minimize fatigue from the workload and to reduce errors regarding evaluation time.

### Statistical analysis

With respect to the groups and evaluation charts, the reliability of measurements was evaluated, while the correlations between the P and S chart items in each group were analyzed using Pearson’s correlation analysis. The measured evaluation time and scores were analyzed using the paired *t*-test. All statistical analyses were performed using IBM SPSS Statistics (Version 26.0. Armonk, NY, USA), with the significance level of p < 0.05.

## Results

### Reliability

As shown in Table 4, the inter-rater reliability for single measures was ≥ 0.87 in all groups and the results were statistically significant (all p < 0.001). The intra-rater reliability for single measures was ≥ 0.72 and ≥ 0.69 in the OMFR and G groups, respectively, and the results were statistically significant (all p < 0.001).

**Table 4:** Results of the reliability analysis

Reliability	Group	Rater	Single measures	*P*
Inter-rater reliability	OMFR	–	0.929	< 0.001
	G	–	0.870	< 0.001
Intra-rater reliability	OMFR	#1	0.867	< 0.001
		#2	0.722	< 0.001
		#3	0.927	< 0.001
		#4	0.926	< 0.001
		#5	0.955	< 0.001
	G	#1	0.694	< 0.001
		#2	0.967	< 0.001
		#3	0.743	< 0.001
		#4	0.827	< 0.001
		#5	0.762	< 0.001

*OMFR* oral and maxillofacial radiologists, *G* general dentists

### Correlation analysis between evaluation items

Table 5 shows the correlation analysis results for all the evaluation items by group (OMFR and G groups) and chart (P and S charts). The results for each group showed that the correlation results were all statistically significant (all p < 0.05). The OMFR group showed very high positive correlations for the total score, coverage area, image density/contrast/sharpness, and the overall image quality grade (all r ≥ 0.804). Meanwhile, the G group showed a relatively lower range of correlation coefficients than the OMFR group; however, the correlation coefficient for the same evaluation items remained high (all r ≥ 0.597). With respect to patient movement, both groups showed low correlation coefficients (r = 0.452, OMFR group; r = 0.405, G group). The results for each chart showed that all the correlation results were statistically significant (all p < 0.05). The P chart showed very high positive correlations for total score, image density/contrast/sharpness, and the overall image quality grade (all r ≥ 0.824). Meanwhile, the S chart showed a relatively lower range of correlation coefficients than the P chart. Similar to the analysis of groups, the lowest correlation coefficient in both charts was for patient movement (r = 0.373, P chart; r = 0.231, S chart).

**Table 5:** Pearson correlation coefficient values (r) of the image evaluation items between each group and evaluation chart

Evaluation item	Group		Chart	
	OMFR	G	P	S
Total score	0.871	0.663	0.884	0.801
Artifacts	0.682	0.416	0.495	0.428
Coverage area	0.820	0.610	0.796	0.434
Patient’s position	0.761	0.548	0.762	0.725
Patient’s movement	0.452	0.405	0.373	0.231
Density, contrast, and sharpness	0.804	0.597	0.824	0.760
Overall image quality	0.817	0.585	0.742	0.741

### Evaluation time and score

Table 6 shows the amount of time the OMFR and G groups spent to complete the evaluation using the P and S charts. In the OMFR group, the evaluation time was 96.67 ± 58.55 s with the P chart and 62.93 ± 50.11 s with the S chart, showing a statistically significant difference of 33.74 between the two charts (t = 60.50, p < 0.001). In the G group, there was a statistically significant difference between the P and S charts (71.13 ± 30.13 s and 45.28 ± 17.61, s respectively; t = 59.07 s, p < 0.001).

**Table 6:** Amount of time [seconds] between the P and S chart assessments in each group

Group	Chart	Mean	SD	SEM	Mean difference	t	*P*
OMFR	P	96.67	58.55	1.16	33.74	60.501	< 0.001
	S	62.93	50.11	0.99			
G	P	71.13	30.13	0.65	25.85	59.068	< 0.001
	S	45.28	17.61	0.33			

Groups (*OMFR* oral and maxillofacial radiologists, *G* general dentists), chart (*P* professional clinical image quality evaluation chart, *S* simple clinical image quality evaluation chart), *SD* standard deviation, *SEM* standard error of the mean

Table 7 shows the scores for the evaluation items measured using the P and S charts by the OMFR and G groups. The G group showed statistically significant differences in all the evaluation items measured using the P and S charts (all p < 0.001). The OMFR group showed statistically significant differences in all the evaluation items (all p < 0.001), except for the coverage area (p = 1.00), patient movement (p = 0.07), and overall image quality grade (p = 0.42). With respect to the mean difference in the measured scores, the highest mean difference was found in the total score in both groups (3.03, OMFR group; 1.37, G group), and for a single item, the highest mean difference was found in image density/contrast/sharpness (2.18, OMFR group; 0.76, G group).

**Table 7:** Comparison of scores between the P and S charts in each group

Group	Evaluation item	Chart	Mean	SD	SEM	Mean difference	*t*	*p*
OMFR	Total score	P	67.30	13.90	0.26	3.03	22.090	< 0.001
		S	64.27	14.74	0.28			
	Artifacts	P	3.27	1.05	0.02	0.18	11.253	< 0.001
		S	3.08	1.14	0.02			
	Coverage area	P	4.93	1.74	0.03	0.00	0.00	1.00
		S	4.93	1.74	0.03			
	Patient’s position	P	14.36	3.19	0.06	0.64	14.725	< 0.001
		S	13.72	3.46	0.07			
	Patient’s movement	P	3.98	0.22	0.004	0.01	1.810	0.07
		S	3.97	0.26	0.005			
	Density, contrast, and sharpness	P	33.74	9.55	0.18	2.18	18.829	< 0.001
		S	31.56	10.08	0.19			
	Overall image quality	P	7.01		0.04	0.02	0.814	0.42
		S	6.00		0.04			
G	Total score	P	67.36	13.33	0.25	1.37	6.270	< 0.001
		S	65.98	14.91	0.28			
	Artifacts	P	3.79	0.68	0.01	0.30	16.079	< 0.001
		S	3.49	1.06	0.02			
	Coverage area	P	5.66	1.07	0.02	0.07	– 3.915	< 0.001
		S	5.72	0.93	0.02			
	Patient’s position	P	14.65	3.06	0.06	0.51	8.766	< 0.001
		S	14.14	3.43	0.06			
	Patient’s movement	P	3.89	0.53	0.01	0.13	9.241	< 0.001
		S	3.79	0.76	0.01			
	Density, contrast, and sharpness	P	32.30	9.91	0.17	0.76	4.247	< 0.001
		S	31.45	11.11	0.21			
	Overall image quality	P	7.08	2.29	0.04	0.25	– 6.507	< 0.001
		S	7.33	2.28	0.04			

Group (*OMFR* oral and maxillofacial radiologists, *G* general dentists), chart (*P* professional clinical image quality evaluation chart, *S* simple clinical image quality evaluation chart), *SD* standard deviation, *SEM* standard error of the mean

Table 8 shows the scores of the evaluation items between the two groups when the items were measured using the P and S charts. When the P chart was used, the results showed statistically significant differences in all evaluation items (all p < 0.001), except for the total score (p = 0.778) and overall image quality grade (p = 0.200). The mean difference in the scores was highest for the image density/contrast/sharpness with 1.43 (33.78, OMFR group; 32.34, G group), while the mean difference for all the other items was < 1. When the S chart was used, there were statistically significant differences in all the evaluation items (all p < 0.001), except for the image density/contrast/sharpness (p = 0.857). In the G group, there were statistically significant differences in all the evaluation items measured using the P and S charts (all p < 0.001). The highest mean difference was found in the total score at 1.68 (64.33, OMFR group; 66.01, G group), while the mean difference for all the other items was < 1.

**Table 8:** Comparison of scores between the groups by chart

Chart	Evaluation item	Group	Mean	SD	SEM	Mean difference	*t*	*p*
P	Total score	P	67.38	8.79	0.37	0.06	– 0.282	0.778
		G	67.44	9.54	0.40			
	Artifacts	P	3.27	0.63	0.03	0.52	– 22.087	< 0.001
		G	3.79	0.43	0.02			
	Coverage area	P	4.96	1.39	0.06	0.71	– 17.715	< 0.001
		G	5.67	0.66	0.03			
	Patient’s position	P	14.36	2.20	0.09	0.29	4.754	< 0.001
		G	14.65	1.97	0.08			
	Patient’s movement	P	3.98	0.11	0.004	0.09	7.574	< 0.001
		G	3.89	0.31	0.01			
	Density, contrast, and sharpness	P	33.78	5.36	0.23	1.43	8.871	< 0.001
		G	32.34	6.77	0.28			
	Overall image quality	P	7.03	1.74	0.07	0.06	– 1.283	0.200
		G	7.09	1.54	0.06			
S	Total score	P	64.33	9.61	0.40	1.68	– 6.424	< 0.001
		G	66.01	10.63	0.45			
	Artifacts	P	3.09	0.70	0.03	0.40	– 14.680	< 0.001
		G	3.49	0.61	0.03			
	Coverage area	P	4.95	1.35	0.06	0.78	– 18.808	< 0.001
		G	5.72	0.59	0.02			
	Patient’s position	P	13.73	2.37	0.10	0.40	– 5.622	< 0.001
		G	14.13	2.31	0.10			
	Patient’s movement	P	3.97	0.12	0.005	0.21	12.182	< 0.001
		G	3.76	0.47	0.02			
	Density, contrast, and sharpness	P	31.60	6.18	0.26	0.04	0.180	0.857
		G	31.56	7.73	0.32			
	Overall image quality	P	7.00	1.73	0.07	0.34	– 7.065	< 0.001
		G	7.34	1.56	0.07			

Group (*OMFR* oral and maxillofacial radiologists, *G* general dentists), chart (*P* professional clinical image quality evaluation chart, *S* simple clinical image quality evaluation chart), *SD* standard deviation, *SEM* standard error of the mean

### Preferences

Table 9 shows the reasons for the preferences between charts. Among a total of 10 evaluators, the preference for the P and S charts was 20% (n = 2; all from the OMFR group) and 80% (n = 8 total; OMFR group, n = 3; G group, n = 5), respectively. The most common reasons why the evaluators in each group preferred the S chart were ease of selecting the evaluation items and time-saving (shorter evaluation time), followed by clarity and simplicity of the evaluation items. None of the evaluators in the G group preferred the P chart.

**Table 9:** Preferences of all raters

	Total		OMFR group		G group	
	P	S	P	S	P	S
Accuracy	1	0	1	0	0	0
Clarity	2	4	2	1	0	3
Detailed	1	0	1	0	0	0
Facility	0	7	0	3	0	4
Simplicity	0	4	0	1	0	3
Time-saving	0	7	0	3	0	4
Others	0	0	0	0	0	0

*n*   number of responses. *OMFR group* oral and maxillofacial radiologists group, *G group *general dentists group, *P* professional clinical image quality evaluation chart, *S* simple clinical image quality evaluation chart

## Discussion

In this study, the correlation analysis results for OMFR and G group showed that the correlation coefficients of the P and S charts for the evaluation items ranged from 0.452 to 0.871 in the OMFR group and 0.405–0.663 in the G group. The findings suggest that the OMFR group performed the quality evaluation of panoramic radiographs with relatively higher consistency. In the analysis by evaluation charts, the correlation coefficients of the OMFR and G groups showed a higher range of 0.373–0.884 with the P chart, as compared to 0.231–0.801 with the S chart. We believe that this is because the P chart had more evaluation items than the S chart and the points assigned were also lower. In the overall evaluation items such as total score, coverage area, image density/contrast/brightness, the S and P charts showed a high correlation, and these results suggest that the S chart can be used with a high confidence. However, the items that showed relatively low correlation, such as artifacts, patient positioning, and patient movement, may need to be improved.

With respect to the evaluation time, both the OMFR and G groups took more time to fill out the P chart than the S chart, which could be attributed to the characteristics of the evaluation items in the P chart (higher number of evaluation items and more detailed evaluation items as compared to the S chart). Especially, image density/contrast/sharpness in the P chart required more detailed evaluation than the same items in the S chart. Moreover, such characteristics of the P chart and differences in the evaluation time were also reflected in the evaluation chart preference survey as the evaluators highly preferred the S chart.

In the evaluation score results, the OMFR group showed statistically significant differences in the coverage area, patient movement, and overall image quality grade among the items measured using the P and S charts. However, the mean difference in each item was small with a minimum of 0.00 (coverage area) and a maximum of 2.18 (density, contrast and sharpness), excepted for total score. The G group showed statistically significant differences in all the evaluation items; however, the mean difference was small with a minimum of 0.07 (coverage area) and a maximum of 0.76 (density, contrast and sharpness), excepted for total score. For coverage area, there was few differences between OMFR and G group, and for density, contrast and sharpness there was few differences between OMFR and G group.

Moreover, when the P chart was used, the differences between the two groups were significant in all the evaluation items, except for the total score and overall image quality grade; however, the mean difference of each group was a minimum of 0.06 (overall image quality) and a maximum of 1.43 (density, contrast and sharpness). When the S chart was used, the differences between the two groups were statistically significant for all the evaluation items; however, the range of the mean difference was small with a minimum of 0.04 (density, contrast and sharpness) and a maximum of 0.78(coverage area), excepted for total score. There was most difference between P and S charts at density/contrast/sharpness Although there were statistically significant differences in the scores, the scores for each evaluation item ranged between 2 and 6 points. The range of the mean difference from a minimum of 0.00 (Table 7, OMFR, coverage area) to a maximum of 2.18 (Table 7, OMFR, density, contrast and sharpness) except for the total score points between the evaluation charts and groups may be somewhat limited to give any important meaning for image evaluation in clinical practice.

In the survey of preference for evaluation charts for panoramic radiography, both the OMFR and G groups preferred the S chart, and the reasons for preferring S chart included ease of selecting the evaluation items and time-saving (shorter evaluation time). This demonstrated that the results from the investigation of the evaluation items showing a shorter evaluation time using the S chart, as compared to the P chart, in the OMFR (33.74 s) and G groups (25.84 s) were also reflected in the preference survey results. The evaluation time was shorter in the G group because the evaluators in the G group reviewed the evaluation items relatively less thoroughly than those in the OMFR group or simply did not spend enough time on the evaluation. Moreover, there are ongoing studies on partial automation of the P chart to upgrade the aforementioned limitations in using the P chart (variety of evaluation items and long evaluation time). Thus, recent studies are actively investigating methods for applying artificial intelligence (AI) [24–26]. Algorithms based on deep learning, a type of AI, are used to apply accumulated radiographic data to effectively detect lesions and segment images, while for quality control of diagnostic radiographic images, some studies have evaluated the feasibility of a manual approach, which can be replaced with system automation [27–30]. The actual clinical application of such results may not fully meet the professional knowledge and expectations of clinicians with respect to ease of use, processing speed, and accuracy [31]. However, if automated clinical image evaluation achieved through technological advances can provide high accuracy and expediency, this can improve the overall quality of diagnostic dental procedures and contribute to patient safety and the prevention of medical accidents. Moreover, the panoramic radiographic data collected in this study were not acquired from the same imaging system, and the evaluation was performed based on a broad range of images acquired by various functions and types of imaging systems used in each hospital. Accordingly, the data acquisition and analysis results in this study have the advantage of ensuring objectivity in the image quality evaluation for panoramic radiography, while use by various evaluators, repeated evaluation, and significant correlation between two clinical image quality evaluation charts can enhance the consistency and reliability of evaluations.

## Conclusions

The S chart, which was developed for frequent and easy use by non-radiologists in dental clinics, can be used to obtain evaluation scores more quickly. However, in order to accurately evaluate image quality, a group of OMFR with a professional evaluation chart would be better. Based on the results of this study, the S chart items that showed low correlations in this study, such as artifacts, coverage area, and patient movement, need to be improved.

## Data Availability

Not applicable.
